# Concentration–effect relationship for tranexamic acid inhibition of tissue plasminogen activator-induced fibrinolysis *in vitro* using the viscoelastic ClotPro® TPA-test

**DOI:** 10.1016/j.bja.2023.09.027

**Published:** 2023-11-03

**Authors:** Christoph Dibiasi, Stefan Ulbing, Dagmar Bancher-Todesca, Martin Ulm, Johannes Gratz, Peter Quehenberger, Eva Schaden

**Affiliations:** 1 Department of Anaesthesia, Intensive Care Medicine and Pain Medicine, Medical University of Vienna, Vienna, Austria; 2 Ludwig Boltzmann Institute Digital Health and Patient Safety, Vienna, Austria; 3 Department of Obstetrics and Gynecology, Medical University of Vienna, Vienna, Austria; 4 Department of Laboratory Medicine, Medical University of Vienna, Vienna, Austria

**Keywords:** ClotPro, fibrinolysis, obstetric haemorrhage, tranexamic acid, viscoelastic testing

## Abstract

**Background:**

Tranexamic acid is an antifibrinolytic drug that is commonly administered for obstetric haemorrhage. Conventional viscoelastic tests are not sensitive to tranexamic acid, but the novel ClotPro® TPA-test can measure tranexamic acid-induced inhibition of fibrinolysis. We aimed to evaluate the TPA-test in pregnant and non-pregnant women.

**Methods:**

We performed an *in vitro* study of whole blood samples spiked with tranexamic acid from pregnant women in the first, second, and third trimester (*n*=20 per group) and from non-pregnant women (*n*=20). We performed ClotPro TPA-tests of whole blood sample and ClotPro EX-tests, FIB-tests, and TPA-tests.

**Results:**

Clot lysis was inhibited in a concentration-dependent manner up to a tranexamic acid concentration of 6.25 mg L^−1^. At tranexamic acid concentrations of 12.5 mg L^−1^ and above, clot lysis was completely inhibited. The concentration–effect relationship of tranexamic acid did not differ in a clinically important manner in blood from pregnant women across all three trimesters or from non-pregnant controls. A median maximum lysis cut-off value of at9 least 16% (25–75th percentiles 15–18), a median clot lysis time of 3600 s (25–75th percentiles 3600–3600), or both was associated with a tranexamic acid concentration of least 12.5 mg L^−1^.

**Conclusions:**

The ClotPro® TPA-test is sensitive in detecting inhibition of fibrinolysis by tranexamic acid in whole blood samples of pregnant and non-pregnant women. The concentration–effect relationship of tranexamic acid to inhibit fibrinolysis in whole blood did not differ for women in the first, second, and third trimester or for non-pregnant women.

**Editor's key points**

• Tranexamic acid is commonly administered for obstetric haemorrhage, but its effect is not measured by conventional viscoelastic tests.
• The authors assessed the novel ClotPro® TPA-test in measuring tranexamic acid-induced inhibition of fibrinolysis in pregnant and non-pregnant women *in vitro.*
• The ClotPro® TPA-test detected concentration-dependent inhibition of fibrinolysis by tranexamic acid in whole blood samples from pregnant and non-pregnant women, which did not differ between pregnant women in the first, second, or third trimester or in non-pregnant women.
• This viscoelastic test might be useful for individualisation of tranexamic acid administration in patients with obstetric haemorrhage.

During pregnancy, physiological haemostasis shifts to a procoagulant state characterised by increased concentration or activity of coagulation factors I, VII, and VIII.^1^, ^2^, ^3^ This is accompanied by reduced activity of anticoagulant factors resulting from reduced protein S and antithrombin activity,^1^^,^^3^^,^^4^ whereas evidence on protein C activity during pregnancy is less clear.^1^^,^^3^^,^^4^ Fibrinolytic activity is also reduced as plasminogen activator inhibitor (PAI)-1 and PAI-2 activity increases during pregnancy.^5^ Taken altogether, pregnancy leads to a procoagulant state that is also detected with global coagulation assays such as thrombin generation,^6^^,^^7^ or viscoelastic assays such as rotational thromboelastometry (ROTEM).^8^ Nevertheless, postpartum haemorrhage (PPH) occurs in 1–3% of all births and causes significant morbidity and mortality.^9^^,^^10^ The antifibrinolytic drug tranexamic acid (TXA) has been shown to reduce bleeding-related mortality when administered for ongoing PPH.^11^ TXA dosing is often standardised and not individualised, which might be due in part to the of lack of monitoring tools for TXA. Although ROTEM or thromboelastography (TEG) can guide haemostatic resuscitation during PPH, they are not sensitive to TXA. However, the novel viscoelastometric ClotPro® TPA-test enables the measurement of antifibrinolysis at the point of care. Previous studies in orthopaedic^12^ and cardiac surgery^13^ patients have shown that the ClotPro TPA-test can detect TXA-induced inhibition of fibrinolysis. We hypothesised that the ClotPro TPA-test can also determine the degree of TXA-induced antifibrinolysis in pregnant women. Given the underlying changes in the coagulation system in pregnancy, we further hypothesised that the concentration–effect relationship of TXA-induced inhibition of fibrinolysis *in vitro* differs in pregnant women compared with non-pregnant women and changes throughout pregnancy. We thus performed an *in vitro* study to determine the concentration–effect relationship for TXA-induced inhibition of clot lysis as measured by the ClotPro TPA-test in pregnant and non-pregnant women.

## Methods

This study was approved by the ethics committee of the Medical University of Vienna (Vienna, Austria; #1279/2021, June 1, 2021). We obtained written informed consent from all study participants.

### Study procedures

We recruited 60 pregnant women from the Department of Obstetrics and Gynecology at the General Hospital of Vienna, a tertiary academic hospital in Vienna, Austria. We also recruited 20 healthy, non-pregnant female volunteers. None of the study participants had a hereditary or acquired coagulation disorder, and none took any anticoagulant drugs. After obtaining informed consent, we drew blood from an antecubital vein using a 21-gauge butterfly needle (Greiner Bio-One GmbH, Kremsmünster, Austria) with a tourniquet. Blood was drawn into 3.2% citrate and EDTA tubes (Greiner Bio-One GmbH). Aliquots of the blood samples were analysed at the Department of Laboratory Medicine to obtain haemoglobin concentration (reference range for non-pregnant women: 120–160 g L^−1^), platelet count (reference range for non-pregnant women: 1.50–3.50×10^11^ L^−1^), Owren's prothrombin time (reference range for non-pregnant women: 24.6–32.7 s), activated partial thromboplastin time (reference range for non-pregnant women: 27–41 s), and Clauss' fibrinogen concentration (reference range for non-pregnant women: 2–4 g L^−1^). Haemoglobin concentration and platelet count were analysed using a Sysmex XE-2100 cell counter (Sysmex Austria, Vienna, Austria). Prothrombin time, partial thromboplastin time, and fibrinogen were determined using the STA R Max 2 coagulometer (Diagnostica Stago S.A.S., Asnières sur Seine Cedex, France).

We conducted viscoelastic measurements using the commercially available viscoelastic analyser ClotPro® (enicor GmbH, Munich, Germany) according to the manufacturer's instructions. The measurement principle of the ClotPro device is similar to ROTEM and TEG.^12^ In brief, coagulation is initiated in citrated whole blood samples by recalcification and the addition of either tissue factor (EX-test), tissue factor and cytochalasin (FIB-test), or tissue factor and tissue plasminogen activator (tPA; TPA-test) with a final tPA concentration of 650 ng mL^−^^1^.^14^ Changes in sample viscoelasticity are measured for 1 h as clot amplitude (mm) over time (s). The resulting curve can be examined visually, and the following parameters are extracted by the ClotPro software: clotting time (CT [s]; defined as duration from starting the test until an amplitude of 2 mm is reached), clot formation time (CFT [s]; defined as duration from reaching 2 mm amplitude until 20 mm amplitude is measured), amplitude at 5, 10, 20, and 30 min after reaching an amplitude of 2 mm (A5, A10, A20, and A30 [all in mm]), maximum clot firmness (MCF [mm]; i.e. the maximum clot amplitude), clot lysis index at 30 min (CLI30 [%]; i.e. the relative change of clot amplitude at 30 min after an amplitude of 2 mm has been reached in relation to MCF), maximum lysis (ML [%]; i.e. the ratio of minimum to maximum amplitude), and lysis time (LT [s]; i.e. the duration until measured amplitude is below 2 mm). We performed the ClotPro EX-test, FIB-test, and TPA-test of native, unaltered aliquots of blood samples from all study participants. ClotPro EX-test and FIB-test were run for 3600 s and ClotPro TPA-test was run until amplitude was below 2 mm, indicating complete fibrinolysis, or for 3600 s.

To investigate the concentration–effect relationship of TXA-induced inhibition of fibrinolysis, we spiked whole blood sample aliquots with TXA (Medicamentum Pharma GmbH, Allerheiligen im Mürztal, Austria) by mixing 395 μl blood with 5 μl of diluted TXA stock solutions to yield TXA final concentrations of: 0.392, 0.785, 1.57, 3.13, 6.25, 12.5, 25, and 50 mg L^−1^. We performed ClotPro TPA-tests for each TXA-spiked sample within a maximum of 4 h after blood collection (performed by CD, SU).

### Statistical analysis

We performed descriptive statistical analysis, calculating median and 25–75th percentiles for all numerical variables. Differences between all patient groups (first, second, and third trimester and non-pregnant women) were tested using Kruskal–Wallis rank sum tests, and differences between two specific patient groups were tested using Wilcoxon rank sum tests. We calculated Pearson's correlation coefficients to assess the correlation between continuous variables (e.g. between ClotPro parameters and laboratory values). We modelled the concentration–effect relationship between TXA and ClotPro TPA-test parameters LT and ML with two four-parameter log-logistic dose–response models (DRM):

$$f\left(x,\left[b,c,d,e\right]\right)=c+\frac{d-c}{\left(1+\exp\left[b\cdot \left(\log\left[x\right]-\log\left[e\right]\right)\right]\right)}$$

Here, *x* refers to the independent variable (TXA concentration), *b* denotes the steepness of the dose–response curve, *c* and *d* denote the lower and upper asymptotes, respectively, and *e* is the effective dose at a response level of 50% (ED_50_).^15^ For LT, we fixed the upper asymptote at 3600 s, estimated the lower asymptote for all subjects together, and estimated steepness and ED_50_ for each pregnancy group individually. For ML, we estimated lower and upper asymptotes for all subjects together, and estimated steepness and ED_50_ individually for each pregnancy group. We performed logistic regression to determine the cut-off points of TPA-test ML and TPA-test LT that predicted TXA concentrations of at least 12.5 mg L^−1^, which we considered a clinically relevant concentration. We considered those cut-off values optimal that maximised Youden's index. We calculated the median cut-off with 25–75th percentiles, and sensitivity and specificity with 95% confidence intervals (CIs) using bootstrapping with 1000 repetitions. We performed correction for multiple testing using Holm's method and considered *P*<0.05 as statistically significant. We did not perform a formal sample size calculation for this study but based the sample size of *n*=20 per group on our experience with *in vitro* experiments involving viscoelastic coagulation measurements. We used R version 4.2.3 (R Foundation for Statistical Computing, Vienna, Austria)^16^ with the *cutpointr* package^17^ and the *drc* package^15^ for all calculations.

## Results

**Fig 1:**
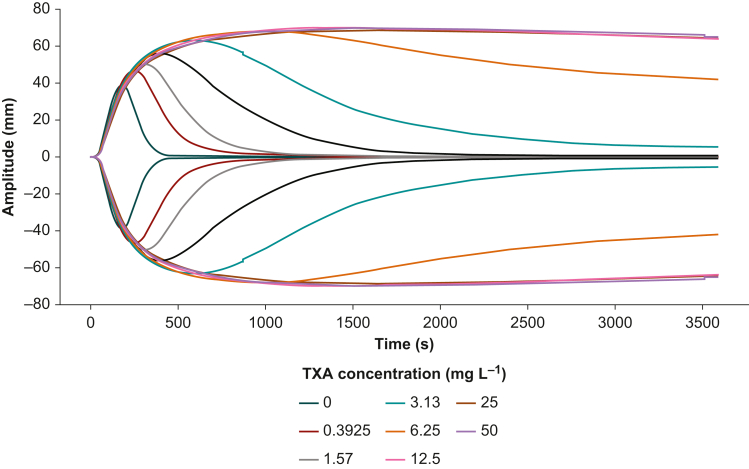
Averaged ClotPro TPA-test results from the tranexamic acid dilution experiments showing increased clot amplitude and reduced clot lysis with higher tranexamic acid concentration. Tranexamic acid concentrations of at least 12.5 mg L^–1^ completely inhibited tissue plasminogen activator-induced lysis.

We performed ClotPro measurements in a total of 80 women, 20 in the first trimester, 20 in the second trimester, 20 in the third trimester, and 20 non-pregnant controls. Median gestational age in the three pregnant patient cohorts was 13 (12–13), 24 (20–24), and 33 (31–35) weeks. Baseline haematological and coagulation laboratory parameters differed between non-pregnant controls and pregnant women across the first, second, and third trimesters (Table 1 and Supplementary Fig. S1). The ClotPro TPA-test results are summarised in Table 2. Samples without TXA showed rapid clot lysis, whereas TXA-spiked samples exhibited concentration-dependent inhibition of clot lysis up to a concentration of 6.25 mg L^−1^. TXA concentrations of 12.5 mg L^−1^ and above completely inhibited clot lysis (Fig. 1).

**Table 1:** Baseline haematological and coagulation parameters. Data are presented as median (25–75th percentiles). *P*-values were obtained using the Kruskal–Wallis rank sum test.

	Non-pregnant	First trimester	Second trimester	Third trimester	*P*-value
Age (yr)	27 (25–30)	33 (28–36)	32 (30–37)	32 (27–35)	0.12
Gestational age (weeks)	-	13 (12–13)	24 (20–24)	33 (31–35)	<0.001
Haemoglobin (g L^−1^)	134 (127–140)	126 (122–129)	110 (106–118)	121 (114–125)	<0.001
Platelet count (10^11^ L^−1^)	2.94 (2.53–3.16)	2.38 (2.37–2.61)	2.26 (2.08–2.34)	2.15 (1.68–2.39)	0.002
Fibrinogen (g L^−1^)	2.49 (2.30–2.66)	3.79 (3.58–4.02)	4.43 (4.33–4.61)	4.91 (4.73–5.53)	<0.001
Prothrombin time (s)	27.1 (27.1–28.6)	27.6 (26.6–28.5)	27.8 (27.4–28.7)	27.0 (26.6–28.1)	0.4
Activated partial thromboplastin time (s)	36.5 (34.1–37.3)	32.7 (31.9–35.9)	34.6 (32.4–36.0)	32.1 (30.8–34.0)	0.007

**Table 2:** ClotPro TPA-test results for whole blood samples spiked with tranexamic acid (TXA). Data are presented as median (25–75th percentiles). *P*-values to test for differences between the four pregnancy groups were obtained using the Kruskal–Wallis rank sum test and adjusted using Holm's method. No *P*-values are given for comparison of lysis time in the samples spiked with TXA to a final concentration of 12.5, 25, and 50 mg L^−1^, as lysis times were numerically identical in all samples.

TXA (mg L^−1^)	Maximum lysis (%)					Lysis time (s)				
	Non-pregnant	First trimester	Second trimester	Third trimester	*P*-value	Non-pregnant	First trimester	Second trimester	Third trimester	*P*-value
0	94 (94–95)	96 (95–96)	96 (96–97)	96 (95–96)	<0.001	195 (172–222)	215 (204–249)	240 (212–263)	206 (192–242)	0.056
0.392	96 (96–96)	96 (95.5–96)	96 (96–97)	97 (97–97)	<0.001	369 (331–424)	323 (288–384)	325 (275–371)	415 (333–511)	0.039
0.785	96 (96–97)	96 (96–97)	97 (96–97)	97 (97–97)	0.200	506 (449–601)	419 (373–520)	443 (381–499)	509 (395–637)	0.077
1.57	97 (97–97)	97 (97–97)	97 (97–97)	97 (97–97)	0.200	988 (784–1070)	633 (584–861)	610 (501–791)	867 (570–1070)	0.009
3.13	97 (87–97)	97 (97–97)	97 (97–98)	97 (97–98)	0.038	1827 (1478–2556)	1143 (823–1732)	1093 (1010–1371)	1174 (909–1501)	0.006
6.25	16 (9–25)	42 (25–69)	34 (23–82)	35 (22–68)	0.002	3600 (3600–3600)	3600 (2615–3600)	3600 (2543–3600)	3600 (2767–3600)	0.150
12.5	6 (5–9)	8 (6–13)	11 (8–13)	8 (4–12)	0.110	3600 (3600–3600)	3600 (3600–3600)	3600 (3600–3600)	3600 (3600–3600)	-
25	6 (4–8)	8 (5–10)	8 (5–10)	5 (3–8)	0.200	3600 (3600–3600)	3600 (3600–3600)	3600 (3600–3600)	3600 (3600–3600)	-
50	6 (4–7)	8 (5–9)	8 (5–10)	3 (3–7)	0.200	3600 (3600–3600)	3600 (3600–3600)	3600 (3600–3600)	3600 (3600–3600)	-

**Fig 2:**
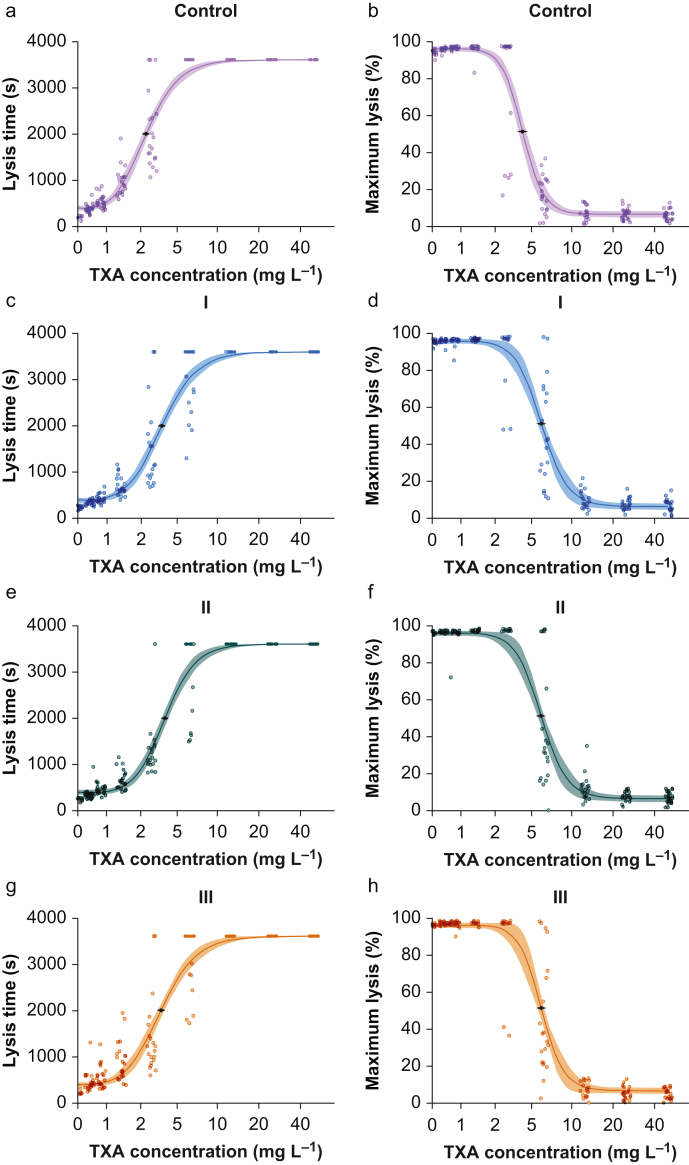
Concentration–effect curve for tranexamic acid (TXA) effect on ClotPro TPA-test lysis time (LT; panels a, c, e, g) and maximum lysis (ML; panels b, d, f, h) for each pregnancy group separately (non-pregnant: panels a and b, first trimester: panels c and d, second trimester: panels e and f, third trimester: panels g and h).

The concentration–effect dependence for TXA-induced inhibition of fibrinolysis as determined by the ClotPro TPA-test is shown in Figure 2. The ED_50_ to prolong LT was 2.8 mg L^−1^ (95% CI 2.6–3.0) in non-pregnant women, 3.8 mg L^−1^ (95% CI 3.5–4.0) in the first trimester, 4.0 mg L^−1^ (95% CI 3.8–4.2) in the second trimester, and 3.7 mg L^−1^ (95% CI 3.5–4.0) in the third trimester. The ED_50_ was statistically different between non-pregnant and pregnant women regardless of trimester, but did not differ statistically between the three trimesters (*P*<0.001 for non-pregnant *vs* first trimester, non-pregnant *vs* second trimester, and non-pregnant *vs* third trimester, *P*=0.335 for first trimester *vs* second trimester, *P*=0.786 for first trimester *vs* third trimester, *P*=0.292 for second trimester *vs* third trimester). The ED_50_ to reduce maximum clot lysis was 4.2 mg L^−1^ (95% CI 4.0–4.6) in non-pregnant women, 6.0 mg L^−1^ (95% CI 5.6–6.3) in the first trimester, 5.9 mg L^−1^ (95% CI 5.6–6.3) in the second trimester, and 5.9 mg L^−1^ (95% CI 5.6–6.2) in the third trimester. The ED_50_ for reduction of ML also differed significantly between non-pregnant and pregnant women, but not within the three trimesters (*P*<0.001 for non-pregnant *vs* first trimester, non-pregnant *vs* second trimester, and non-pregnant *vs* third trimester, *P*>0.999 for first trimester *vs* second trimester, *P*>0.999 for first trimester *vs* third trimester, *P*>0.999 for second trimester *vs* third trimester). Estimated parameters of both DRMs are provided in Supplementary Tables S1 and S2.

We determined cut-off values for ML and LT associated with TXA levels of at least 12.5 mg L^−1^. The median cut-off value for ML was 16% (15–18) with a sensitivity of 99% (95% CI 97–100) and a specificity of 95% (95% CI 93–97). For LT, the median cut-off value was 3600 s (3600–3600) with a sensitivity of 100% (95% CI 100–100) and a specificity of 86% (95% CI 83–88).

**Fig 3:**
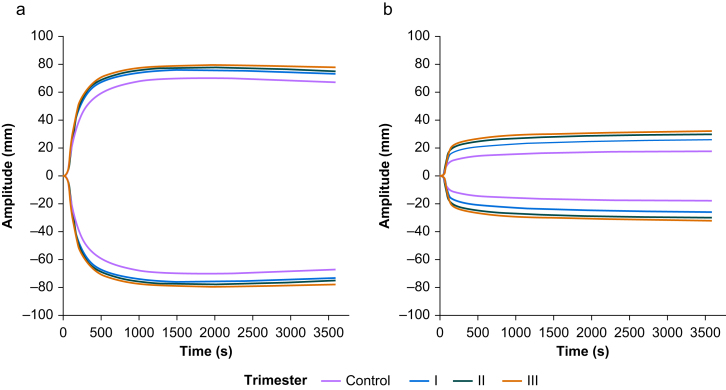
Averaged results from ClotPro EX-test (a) and FIB-test (b) stratified by trimester.

ClotPro EX-test and FIB-test showed increased amplitudes in pregnant *vs* non-pregnant women, whereas CT and CLI30 stayed the same (Table 3, Fig. 3, and Supplementary Figs. S2 and S3).

**Table 3:** Results from the ClotPro EX-test and FIB-test measurements. Data are presented as median (25–75th percentiles). *P*-values to test for differences between the four pregnancy groups were obtained using the Kruskal–Wallis rank sum test and adjusted using Holm's method. A5, amplitude 5 min after an amplitude of 2 mm is reached; A10, amplitude 10 min after an amplitude of 2 mm is reached; A20, amplitude 20 min after an amplitude of 2 mm is reached; A30, amplitude 30 min after an amplitude of 2 mm is reached; CFT, clot formation time; CT, clotting time; MCF, maximum clot firmness.

	Non-pregnant	First trimester	Second trimester	Third trimester	*P*-value
EX-test					
CT (s)	62 (56–68)	63 (57–66)	58 (54–62)	58 (54–62)	0.180
CFT (s)	62 (54–72)	47 (42–52)	47 (42–52)	47 (45–56)	<0.001
A5 (mm)	46 (44–50)	53 (50–56)	54 (51–57)	57 (50–60)	<0.001
A10 (mm)	54 (52–57)	61 (57–64)	62 (59–64)	64 (58–66)	<0.001
A20 (mm)	60 (57–62)	65 (62–68)	66 (63–68)	69 (64–70)	<0.001
A30 (mm)	61 (58–62)	65 (62–68)	66 (65–69)	70 (64–71)	<0.001
MCF (mm)	61 (58–62)	65 (62–68)	66 (65–69)	70 (64–71)	<0.001
CLI30 (%)	100 (100–100)	100 (100–100)	100 (100–100)	100 (100–100)	0.350
FIB-test					
A5 (mm)	11 (10–13)	17 (15–19)	20 (18–22)	22 (16–24)	<0.001
A10 (mm)	12 (11–14)	18 (16–20)	22 (18–24)	24 (18–27)	<0.001
A20 (mm)	13 (12–15)	20 (18–22)	24 (20–26)	26 (20–30)	<0.001
A30 (mm)	14 (13–16)	21 (20–24)	24 (21–27)	27 (20–30)	<0.001
MCF (mm)	14 (13–16)	22 (20–25)	26 (22–28)	28 (22–32)	<0.001
CLI30 (%)	100 (100–100)	100 (100–100)	100 (100–100)	100 (100–100)	0.420

Neither EX-test A5, EX-test A10, nor EX-test MCF correlated with platelet count (A5: r=–0.08, 95% CI –0.35–0.2, *P*=0.562; A10: r=–0.1, 95% CI –0.37–0.18, *P*=0.483; MCF: r =–0.08, 95% CI –0.34–0.2, *P*=0.595; Supplementary Fig. S4). FIB-test A5, FIB-test A10, and FIB-test MCF showed excellent correlation with fibrinogen concentration (A5: r=0.93, 95% CI 0.86–0.96, *P*=<0.001; A10: r=0.93; 95% CI 0.87–0.97; *P*=<0.001; MCF: r=0.94, 95% CI 0.88–0.97, *P*=<0.001; Supplementary Fig. S4).

## Discussion

We applied the ClotPro® TPA-test to determine the concentration–effect relationship for TXA-induced inhibition of fibrinolysis. The ClotPro TPA-test parameters ML and LT were sensitive for detecting inhibition of fibrinolysis induced by different TXA concentrations in whole blood samples. ClotPro EX-test and FIB-test showed pregnancy-related hypercoagulability, as evidenced by increased clot amplitudes. The TXA concentration–effect relationship in inhibiting fibrinolysis was not different between the three trimesters or between pregnant and non-pregnant women.

TXA exerts its antifibrinolytic properties by competitive inhibition of plasminogen, thereby preventing its conversion to plasmin.^18^ TXA administration has been associated with reduced blood loss in obstetric^19^ and gynaecological^20^ surgery. The effective dose of TXA has been estimated to be 10–15 mg L^−1^, but plasma concentrations of at least 5 mg L^−1^ partially inhibit fibrinolysis.^21^ In the setting of PPH, 1 g of TXA should be given i.v. according to World Health Organisation guidelines,^22^ a dose that results in peak plasma concentrations of ∼60 mg L^−1^.^23^ Oral and i.m. administration of TXA also leads to plasma concentrations considered effective.^24^, ^25^, ^26^ Although measuring TXA concentration is feasible in scientific studies, it is not possible in clinical practice, as laboratory coagulation tests and conventional viscoelastic measurements can only indirectly depict fibrinolysis. tPA-modified ROTEM^27^ and plasmin-spiked TEG assays^28^ have been described; however, the ClotPro TPA-test is the first commercially available viscoelastic assay to directly visualise inhibition of fibrinolysis.

A close correlation between TPA-test ML and LT after *in vivo* administration of TXA has been shown in orthopaedic and cardiac surgery patients.^12^^,^^13^ In this study, we showed that the ClotPro TPA-test is also valid in pregnant women, a population in which distinct alterations of haemostasis occur. Pregnancy-related hypercoagulability results from the increased activity of procoagulant factors in combination with reduced anticoagulant and fibrinolytic activity that develops over the course of pregnancy.^29^ Several studies have shown that earlier generations of viscoelastic coagulometers can detect pregnancy-related hypercoagulability,^30^, ^31^, ^32^, ^33^ and we show that the ClotPro EX-test and FIB-test also measure increased clot amplitudes in pregnant compared with non-pregnant volunteers. We have shown that ClotPro and ROTEM measurements highly correlate with each other, but absolute differences in measured clotting times and amplitudes do exist between those devices.^34^ As such, care must be taken in using diagnostic or therapeutic thresholds derived from ROTEM when using ClotPro, and we recommend that future studies establish formal ClotPro reference ranges for pregnant women. With regard to the TPA-test, we provide cut-offs with high sensitivity and specificity to detect TXA concentrations previously described as effective for inhibition of fibrinolysis.

Importantly, how our results are translatable to patients with obstetric haemorrhage and possible hyperfibrinolysis still needs to be determined. tPA is present at a fixed concentration in the TPA-test pipette tip, and it is unclear how this concentration reflects systemic and local fibrinolysis in obstetric bleeding. As such, ClotPro TPA-test results indicating complete inhibition of fibrinolysis *ex vivo* do not necessarily imply that this is true *in vivo*. However, it is likely that in patients with ongoing bleeding, insufficient inhibition of fibrinolysis, evident as rapid clot lysis shown by the ClotPro TPA-test, following the recommended dose of TXA would indicate hyperfibrinolysis, and this finding could prompt clinicians to administer additional TXA doses. Thus, the ClotPro TPA-test could potentially help to individualise TXA treatment in bleeding patients. For example, it could be insightful to repeat the TPA-test 2.5–3 h after the initial administration of TXA given that its plasma concentration falls from 60 to 11.5 mg L^−1^ in this time span.^23^ Although, based on our results, the ClotPro TPA-test is highly sensitive for detecting TXA-induced inhibition of fibrinolysis, viscoelastic measurements in general are poor methods for diagnosing disorders of the fibrinolytic system.^35^ Clot amplitude reductions characteristic of systemic hyperfibrinolysis are not commonly seen during PPH^36^^,^^37^ and can be caused by platelet retraction.^38^ Viscoelastic measurements from vaginal, uterine, or both whole blood samples from patients with PPH have been shown to indicate ineffective coagulation, possibly resulting from *local* hyperfibrinolysis.^39^ Other global assays of fibrinolysis, such as plasmin generation, could be helpful to further elucidate the pathophysiologic importance of fibrinolysis in obstetric bleeding and how TXA alters it.^40^^,^^41^ Plasmin generation can distinguish between hypofibrinolysis and hyperfibrinolysis in patients with traumatic coagulopathy^42^ and is highly sensitive for detecting TXA-induced inhibition of fibrinolysis in patients with Caesarean delivery.^41^^,^^43^ However, in contrast to viscoelastic tests, plasmin generation is currently unavailable in clinical practice.

The most important limitation of our study is the recruitment of healthy study participants without coagulopathy, which could hinder direct application of our findings to clinical practice. Secondly, our *in vitro* experiments cannot be used to infer sufficient inhibition of fibrinolysis *in vivo*, as outlined above. Thirdly, we did not obtain clinical outcomes, such as bleeding, thrombosis, or both because of our study design, but this is ultimately the most important information when using diagnostic tests. Lastly, we did not measure fibrinolytic pathway proteins, such as thrombin-activatable fibrinolysis inhibitor PAI-1, PAI-2, or α_2_-antiplasmin. We can therefore provide no information on whether the ClotPro TPA-test is suitable for detecting alterations in physiological pathways of fibrinolysis.

In summary, we found the ClotPro® TPA-test to be highly sensitive for detecting TXA-induced inhibition of fibrinolysis throughout pregnancy. We did not observe a clinically important change in the *in vitro* TXA concentration–effect relationship between pregnant and non-pregnant women. Future studies should address how the ClotPro TPA-test can be used for individualisation of TXA administration in patients with obstetric haemorrhage.

## Authors’ contributions

Study design: CD, ES

Patient recruitment: CD, SU, DB, MU

Coagulation experiments: CD, SU, PQ

Data analysis and interpretation: CD, SU, JG, ES

Initial draft of the manuscript: CD

Manuscript revision: all authors

## Declarations of interest

CD and SU have received speaking fees from CSL Behring, Astra Zeneca, Arjo, Biomedica, ekomed, NovoNordisk, and Roche. JG has received honoraria, research funding, and travel reimbursement from Alexion, Boehringer Ingelheim, CSL Behring, Instrumentation Laboratory, Johnson & Johnson, Mitsubishi Tanabe Pharma, Octapharma, Portola, and Takeda. ES has received speaking fees from CSL Behring, Astra Zeneca, Arjo, Biomedica, ekomed, NovoNordisk, Roche, B. Braun, and Bristol Myers Squibb. The other authors declare that they have no conflicts of interest.
