# Supplementary material for: Concentration–effect relationship for tranexamic acid inhibition of tissue plasminogen activator-induced fibrinolysis in vitro using the viscoelastic ClotPro® TPA-test
Source: Br J Anaesth. 2023 Nov 3;132(2):343–51. doi: 10.1016/j.bja.2023.09.027 (PMC10808820; doi:10.1016/j.bja.2023.09.027)

**Dose-response relationship of tranexamic acid to inhibit tissue plasminogen activator-induced fibrinolysis: an in vitro study using the viscoelastic ClotPro TPA-test**

**Supplementary Material**

[Supplementary Table 1 2](#_Toc143453291)

[Supplementary Table 2 3](#_Toc143453292)

[Supplementary Figure 1 4](#_Toc143453293)

[Supplementary Figure 2 5](#_Toc143453294)

[Supplementary Figure 3 6](#_Toc143453295)

[Supplementary Figure 4 7](#_Toc143453296)

## Supplementary Table 1

Estimated parameters of the dose-response model for the relationship between tranexamic acid concentration and ClotPro TPA-test lysis time. Steepness and ED50 were estimated for each pregnancy group individually. Lower asymptote was estimated for all samples at a whole. Upper asymptote was fixed at 3600 seconds and not estimated. ED50: effective dose to prolong ClotPro TPA-test lysis time by 50%. CI: confidence interval

|  | **Steepness (-)** | **Lower asymptote (s)** | **Upper asymptote (s)** | **ED50 (mg L^-1^)** |
| --- | --- | --- | --- | --- |
| **Non-pregnancy** | -3.06 (95% CI -3.59–-2.52) | 396 (95% CI 344–448) | - | 2.78 (95% CI 2.59–2.97) |
| **1^st^ trimester** | -3.07 (95% CI -3.61–-2.53) |  |  | 3.77 (95% CI 3.52–4.01) |
| **2^nd^ trimester** | -3.55 (95% CI -4.2–-2.91) |  |  | 4.00 (95% CI 3.76–4.25) |
| **3^rd^ trimester** | -2.98 (95% CI -3.58–-2.38) |  |  | 3.72 (95% CI 3.48–3.97) |

## Supplementary Table 2

Estimated parameters of the dose-response model for the relationship between tranexamic acid concentration and ClotPro TPA-test maximum lysis. Steepness and ED50 were estimated for each pregnancy group individually. Lower and upper asymptotes were estimated for all samples at a whole. ED50: effective dose to reduce ClotPro TPA-test maximum lysis by 50%. CI: confidence interval

|  | **Steepness (-)** | **Lower asymptote (s)** | **Upper asymptote (s)** | **ED50 (mg L^-1^)** |
| --- | --- | --- | --- | --- |
| **Non-pregnancy** | 5.20 (95% CI 4.12–6.27) | 6 (95% CI 5–8) | 96 (95% CI 94–97) | 4.25 (95% CI 3.95–4.55) |
| **1^st^ trimester** | 4.37 (95% CI 2.92–5.83) |  |  | 5.96 (95% CI 5.61–6.31) |
| **2^nd^ trimester** | 4.44 (95% CI 2.92–5.95) |  |  | 5.92 (95% CI 5.59–6.26) |
| **3^rd^ trimester** | 5.11 (95% CI 2.94–7.28) |  |  | 5.93 (95% CI 5.61–6.25) |

## Supplementary Figure 1

Laboratory parameters (haemoglobin, platelet count, fibrinogen concentration, prothrombin time and partial thromboplastin time) stratified by pregnancy group (non-pregnancy, 1^st^ trimester, 2^nd^ trimester, 3^rd^ trimester). APTT: activated partial thromboplastin time.


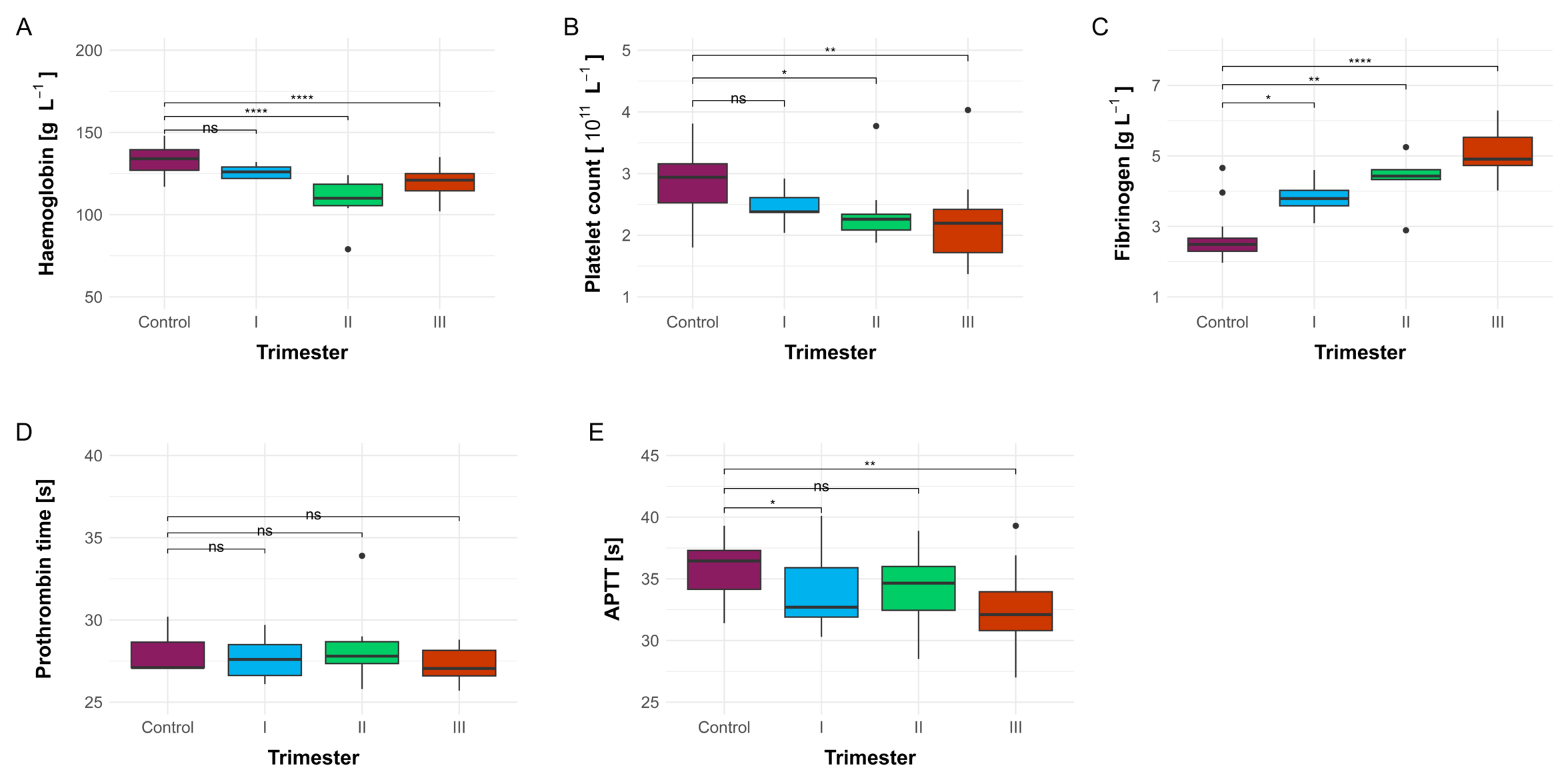


## Supplementary Figure 2

ClotPro EX-test parameters stratified by pregnancy group (non-pregnancy, 1^st^ trimester, 2^nd^ trimester, 3^rd^ trimester). CT: clotting time. CFT: clot formation time. A5: Amplitude 5 minutes after an amplitude of 2 mm is reached. A10: amplitude 10 minutes after an amplitude of 2 mm is reached. A20: amplitude 20 minutes after an amplitude of 2 mm is reached. MCF: maximum clot firmness.


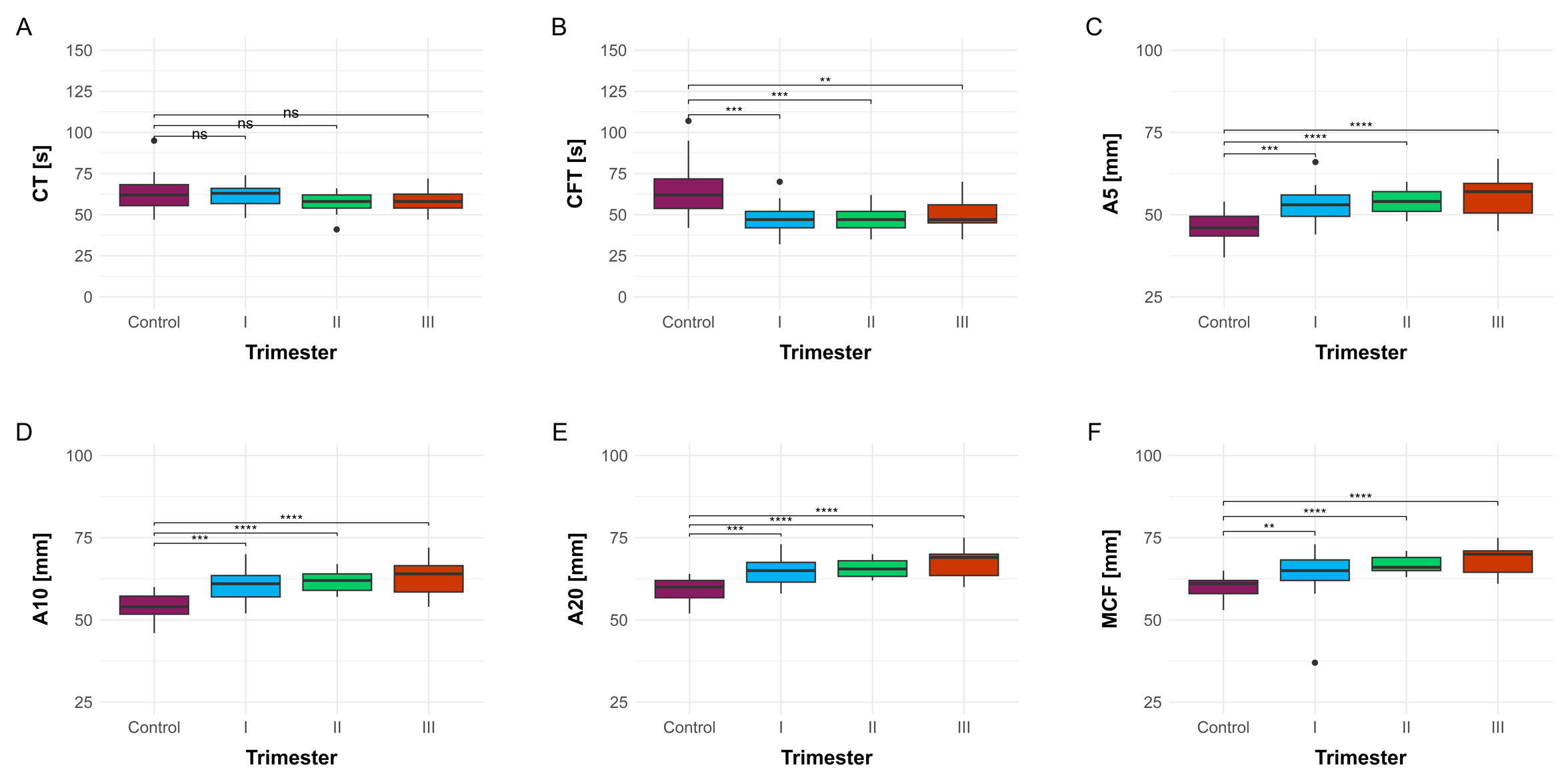


## Supplementary Figure 3

ClotPro FIB-test parameters stratified by pregnancy group (non-pregnancy, 1^st^ trimester, 2^nd^ trimester, 3^rd^ trimester). A5: Amplitude 5 minutes after an amplitude of 2 mm is reached. A10: amplitude 10 minutes after an amplitude of 2 mm is reached. A20: amplitude 20 minutes after an amplitude of 2 mm is reached. MCF: maximum clot firmness.


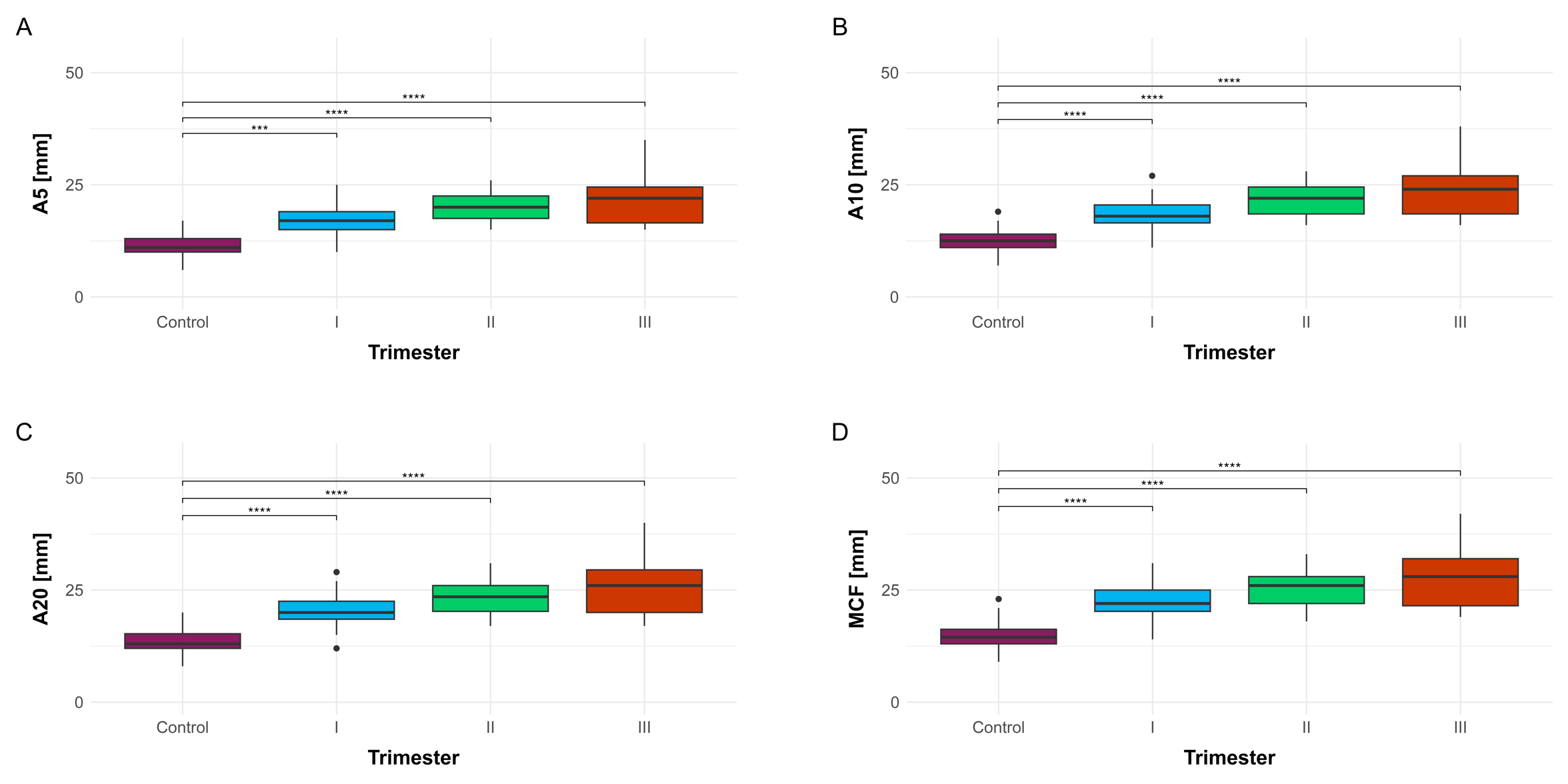


## Supplementary Figure 4

Scatter plots showing the relationship between platelet count with ClotPro EX-Test A5 (panel A), EX-test A10 (panel B) and EX-Test MCF (panel C) as well as fibrinogen concentration with ClotPro FIB-test A5 (panel D), FIB-Test A10 (panel E) and FIB-Test MCF (panel F). A5: Amplitude 5 minutes after an amplitude of 2 mm is reached. A10: amplitude 10 minutes after an amplitude of 2 mm is reached. A20: amplitude 20 minutes after an amplitude of 2 mm is reached. MCF: maximum clot firmness.


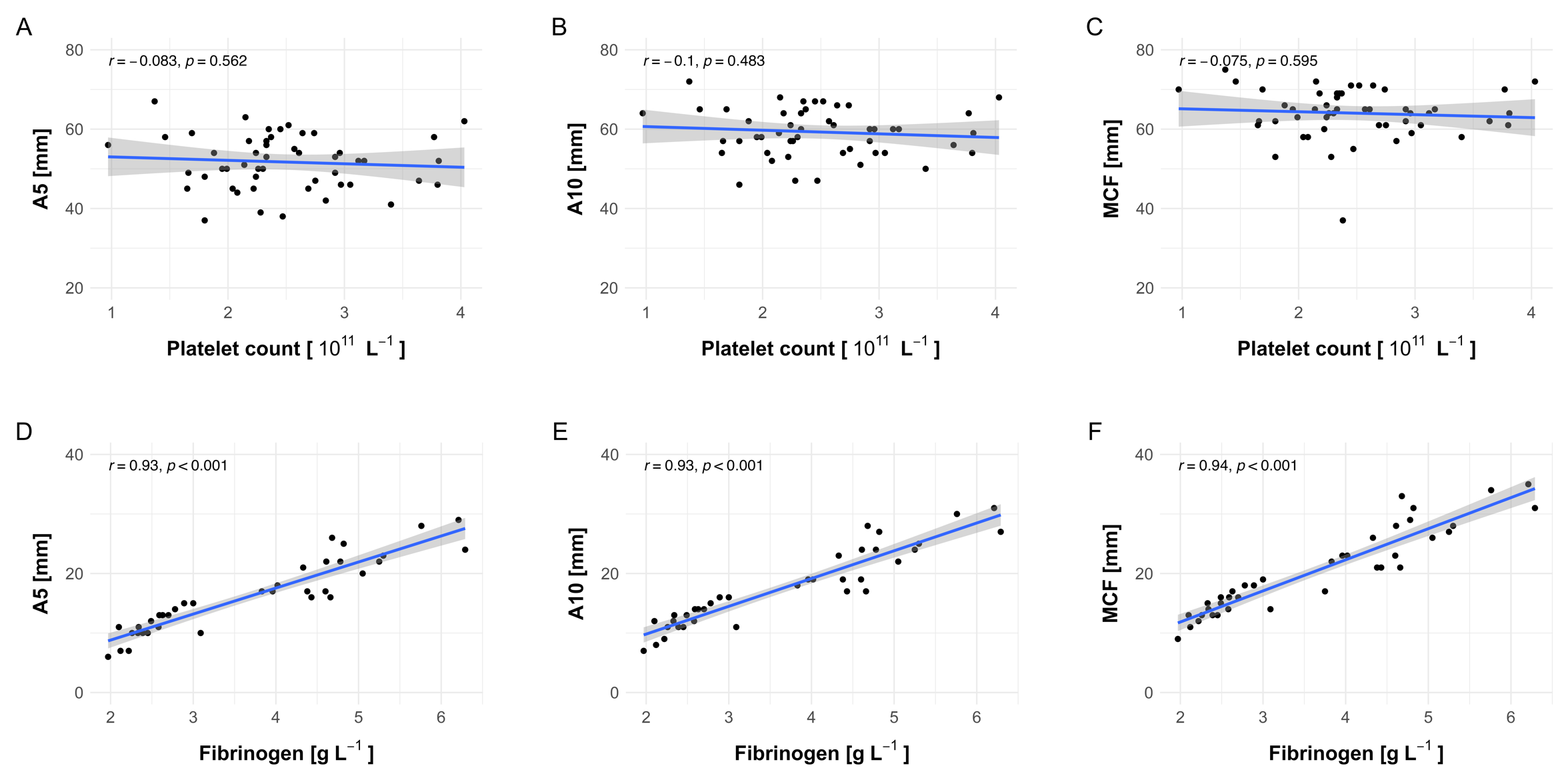

Supplement: Multimedia component 1 [file mmc1.docx]
